# Assessing local people's perceptions of ecosystem services to support land management plans in arid desert regions, northwest China

**DOI:** 10.1016/j.heliyon.2024.e25302

**Published:** 2024-01-26

**Authors:** Qi Tan, Siru A, Wenying Lang

**Affiliations:** a Center for Studies of Ethnic Minorities in Northwest China, Lanzhou University, Lanzhou 730000, Gansu,China; b College of Tourism and Geographical Science, Leshan Normal University, Leshan 614006, Sichuan, China

**Keywords:** Ecosystem services, Arid desert regions, Stakeholder, Land use change, Sustainable development

## Abstract

Deserts play a significant role in terrestrial ecosystems, but their importance is often underestimated in research due to the undervaluation of ecosystem services (ES), particularly the absence of environmental stakeholder perspectives. This study utilized the social science research methodology to investigate the identification and perceptions of desert ES, and the perceived changes in the significance and accessibility of these services following land use alteration among two groups with distinct livelihood strategies in the arid desert region of Northwest China. The study identified 28 ES; with water being the top priority for all; herbs, water and fodder were considered significantly reduced by 78.69 %, 55.74 % and 50.82 % of the PPG, while 62.69 %, 37.31 % and 32.84 % of the APG considered herbs, sense of belonging and link to ancestors to be significantly decreased. The research also explored the potential of social science research methods for assessing ES and contributing to understanding environmental stakeholders' needs and perceptions. It is recommended that future ecological conservation and land project development prioritizes the livelihoods, emotional well-being, and cultural needs of residents. This approach will contribute to the long-term sustainability of both the environment and the project, while also safeguarding the well-being of residents.

## 1 Introduction

Ecosystem services (ES) are the various benefits that humans derive directly or indirectly from ecosystems [1], and are usually subdivided into four main categories: provisioning services, regulating and supporting services, and cultural services [2,3]. Since the release of the United Nations Millennium Assessment Project (MEA), the academic literature studying ES has grown exponentially [4,5]. ES are seen as a tool to communicate and demonstrate the importance of nature as well as biodiversity to human well-being [6,7],and are widely used in policy making and land management [8,9] to weigh and reconcile ecosystem functioning with human well-being, both in public education and in decision making [2,10].

Successful ES assessments are, however, influenced by one key factor: that is, the assessment of ES should be determined by the stakeholders of the environment [[10], [11], [12]]. This is because ES are based on human needs, utilization and preferences and reflect human perception and use of ecosystem functions. Whether humans obtain goods or services from ecosystems depends on human needs and preferences, behaviors, and utilization patterns. Recent research has invested increasing efforts to engage stakeholders in ES research to report their perspectives on ES [13,14]. This can efficiently diminish conflicts between socio-economic progression and the utilization of natural resources. In addition, it is vital for devising environmentally sustainable development blueprints, resolving land-use predicaments, and advancing the welfare of local communities [[15], [16], [17]].

With the growing importance of ES, a variety of valuation methods have been developed to determine the value of ES to support relevant decision-making at regional and national levels [18]. Social science research methods, such as interviews and field observations, are better able to evaluate ES from the perspective of environmental stakeholders, especially in a non-economic way [12,19,20]. They can complement and enhance traditional economic and ecological approaches [21,22], because they explicitly take the stakeholders as the focus of research, allowing for a better understanding of the complex socio-cultural-ecological systems of local communities [23]. Furthermore, using social science research methods can help identify cognitive differences within stakeholder groups, thus exploring trade-offs between stakeholders for ES [24,25], and can be useful in revealing the pros and cons of land-use change and development projects [[26], [27], [28]], and in informing environmental protection and management decision-making processes [29].

Desert arid regions, such as the Gobi and deserts, are vulnerable to temperature extremes and have higher evaporation rates than precipitation [30]. These regions are regarded as having lower biodiversity and lower ES values and have gotten less attention in ES research [31]. Nonetheless, arid and semi-arid desert areas, which are a significant part of terrestrial ecosystems, cover a sizable amount of land; in fact, they make up 42 % of China's national territory alone [32]. Due to the barren natural environment and resource conditions, a high degree of coupling between desert arid regions and socio-economically underdeveloped regions occurs in northwestern China [33]. The Chinese government has been committed to promoting the social and economic development of western China, especially the desert and arid regions in northwest China, through various policies such as the Western Development Program, to lift the people living there out of poverty.

However, in the process of project development and economic development, the conflict between resource utilization and environmental protection has become increasingly prominent. Chinese scholars have begun to reflect on the impact of unbalanced economic and environmental development on ethnic regions from the perspective of ecological ethnology. They have explored and reflected on the relationship between social development, cultural adaptation and local knowledge protection, and have made greater contributions to the protection of traditional local ecological knowledge [34]. Ecological anthropology, as a product of interdisciplinarity, focuses on cultural studies as the disciplinary nature and theoretical characteristics of Chinese ecological anthropology, but lacks the methods and concepts of ecology. Ecological ethnology links the study of human life and the environment, and it happens that ES is the best link to study the relationship between people and the environment [35].

The mature theoretical connotation, rich content and methodological system of ES can be directly applied to the research framework of ecological ethnology, reflecting the degree of matching of supply and demand, spatial patterns and their temporal and spatial changes by establishing quantitative and spatial correlations between the beneficiaries of ES (socio-economic systems) and the providers of ES (natural ecosystems) [36]. Meanwhile, ES have a complete categorization system and quantitative assessment methods, and the evaluation indicators can help people record, describe and measure the ways and quantities of the ecosystems that provide material, economic and cultural well-being for human beings. This attempt is useful in providing more interdisciplinary research methods and ideas for Ecological ethnology in China, which is also the academic significance of this study.

To inform policy development, and to prevent harming the well-being of local people [37], it is important to consider the needs and perspectives of local people. Consequently, it is necessary to understand how local community residents with different livelihood strategies to identify [38], and use ES in desert arid regions, and which ES in desert arid regions would be more valued and prioritized. We are aware of few studies that have employed social science research techniques to determine ES from the perspective of residents in the desert arid regions of northwest China. Our research questions are therefore: (i) How do local community residents identify and perceive ES in arid desert regions. (ii) Which desert ES are valued and prioritized by groups with different livelihood strategies. (iii) The impact of land use change on changes in the perceived availability of ES by groups with different livelihood strategies.

## 2 Materials and methods

### 2.1 Study area

**Fig. 1:**
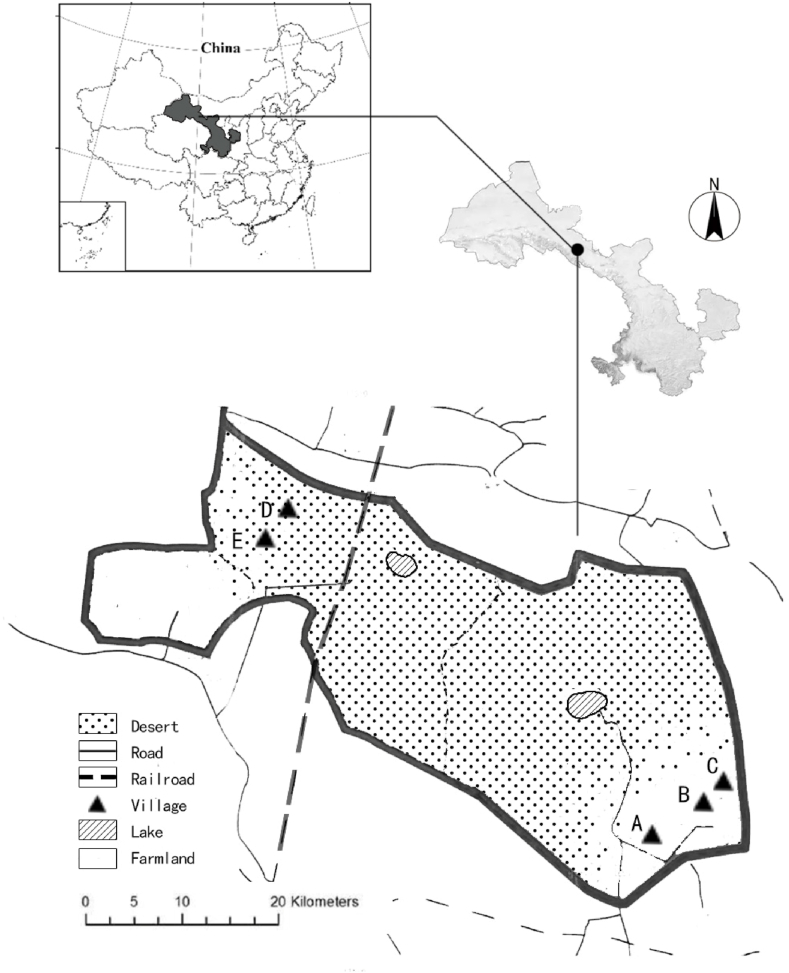
Schematic of the study area.. (After the “Agricultural Settlement Project”, the Yugurs moved from the desert where they called “former home" to settle on the edge for farmland reclamation. Although those who are now engaged in herding still need access to desert pastures, their main settlements are still in agricultural areas.)

This study area is in five villages in the arid inland hinterland of northwest China, in the central Hexi Corridor, on the southwestern edge of the Badanjilin Desert (Fig. 1). The area covers 1704 square kilometers, with an average elevation of 1300–1450 m above sea level, annual precipitation of 66–87 mm, high average annual evaporation of 2000–2900 mm. The average annual temperature is 7.8° Celsius, with an extreme minimum temperature of −30° Celsius and an extreme maximum temperature of 40° Celsius, which is a typical inland desert-type climate. The vegetation cover is less than 20 %, mainly desert-arid grassland, characterized by drought and water scarcity, sparse vegetation, serious salinization, and desertification.

**Fig. 2:**
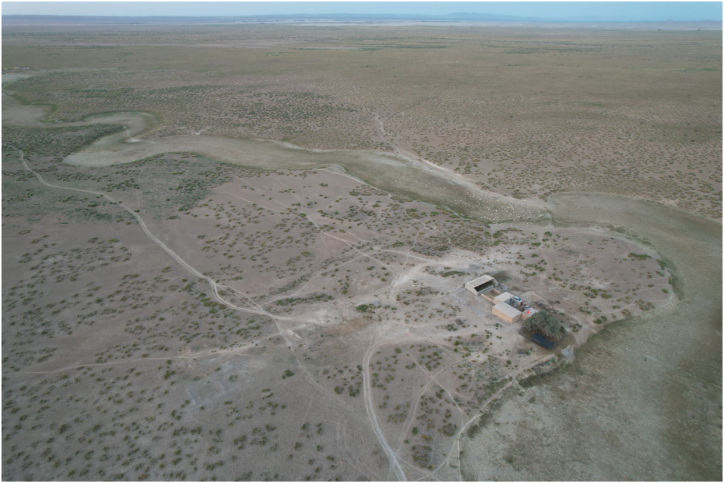
The arid pastures of the desert.. (Taken: July 2023; by T.Q.)

**Fig. 3:**
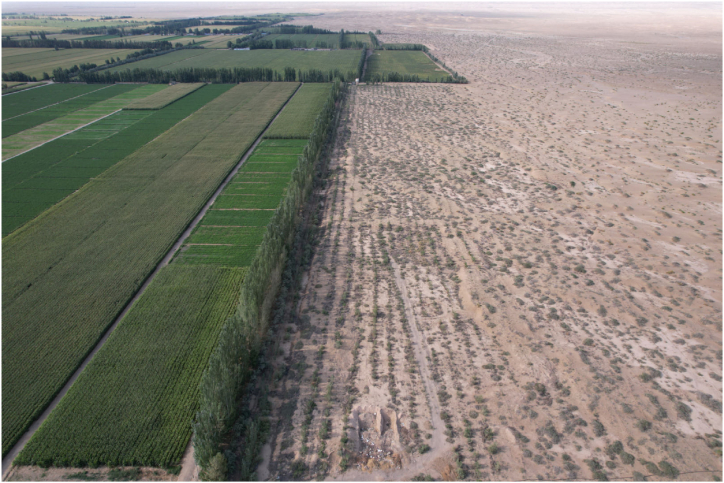
Farmland and windbreaks around agricultural settlements.. (Taken: July 2023; by T.Q.)

Living in these five villages are mainly the Yugurs, a unique ethnic minority in Gansu Province. The Yugurs who dwell here have selected animal husbandry as the most practical means of subsistence in response to the environment for a long period of time to adapt to this unique natural environment (Fig. 2). However, with the growth of population, the expansion of livestock and the decline of water table, the pastureland continued to degrade, seriously affecting and restricting the improvement of the living standard of the local residents. In 1999, in order to completely solve the problem of poverty among the villagers, the local government implemented the “Agricultural Settlement Development Project", which relocated the people who used to live scattered in the desert dry pastures to the designated agricultural development zones (Fig. 3), so as to enable the Yugur people, who had been engaged in pastoral production for generations, to learn farming techniques.

Over the past two decades, the local population has gone through a difficult process of changing and adapting to their way of life. Today, one after another, some of the villagers have returned to the arid pastures of the desert to continue their pastoral production. Others, who have chosen to engage in agricultural production around the village, keep some livestock in captivity around their farmhouses as a meat supply or an economic source. After two decades of choice and differentiation, the villagers have formed two groups with a balance between the two modes of production, but with different livelihood strategies.

In 2021, the resident population of the five villages was 478 and was mainly middle-aged and elderly, while the young people mainly went out to work or study. Usually, they all live in the villages, with those engaged in agriculture farming in the farmlands near the villages and those engaged in pastoralism living in temporary dwellings in the desert pastures from May to September. In none of the five villages are all villagers engaged in agriculture or all villagers engaged in pastoralism, and villagers with different livelihood strategies live intertwined in the five villages. Therefore, this study categorized the respondents into two groups based on their livelihood strategies: those who are mainly engaged in pastoral production and those who are mainly engaged in agricultural production.

(i) Respondents who are predominantly pastoralists (hereafter referred to as PPG for this group), who are mainly characterized by the fact that their labor is mainly invested in pastoral production in desert pastures, and that agricultural production is small-scale or not engaged in agricultural production activities.
(ii) Respondents who are mainly engaged in agricultural production (hereinafter referred to as APG for this group), who are characterized by mainly agricultural production activities, only keeping small livestock around the farmhouse, and do not directly engage in pastoral production activities in the desert arid grassland.

### 2.2 Data Collection and analysis

The research team conducted field surveys in the above areas from July–August 2021, and from July–October 2022, and July–September 2023. The research team was introduced to the residents by the mother of one of the researchers, at a family gathering. Her mother is a very famous Yugur language teacher in the area and is well respected by the villagers. This was very important for the research team to have a smooth entry into the local community and to gain access to the respondents. The few months we lived in the study community were very important in building trust and rapport with the community members.

A total of 128 respondents, including 61 PPGs and 67 APGs, were local villagers and met the criteria for livelihood strategies (Table 1). During the survey, it was found that most young people went out to study or work, and fewer were engaged in agriculture or pastoral production at home.

**Table 1:** The information for respondents.

	Gender		Age			Education level			
	Male	Female	Below 50	51–60 years old	Above 61	Below junior	Junior school	High school	Above high school
PPG	55.74 %	44.26 %	8.96 %	68.66 %	22.39 %	22.95 %	60.66 %	14.75 %	1.64 %
APG	70.15 %	29.85 %	39.34 %	45.90 %	14.75 %	8.96 %	34.33 %	47.76 %	8.96 %

**Fig. 4:**
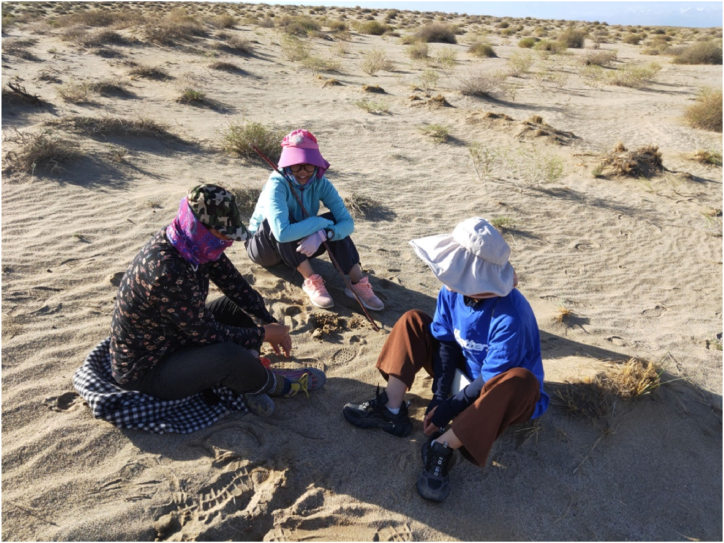
Investigators interviewing residents (1st from left) in the desert (Taken: Aug. 2022; by L.WY).

Based on the above delineation of respondents' livelihood strategies, the team entered the field and first randomly selected respondents for each group for participant observation and structured interviews. The purpose of the study was explained to the respondents to understand how they conduct their productive livelihoods in the desert. Each respondent participated voluntarily, knowing the purpose of the study, and agreed to be audio-recorded during the interviews, as well as to the investigator's involvement in their productive lives during this time to observe how they use ES. The participant-observation research method is one of the common methods of anthropological research, i.e., the researcher goes into the real-life context of the research subjects and observes and records them while actually participating in their daily social life [39]. To gain a deeper understanding of how local community members construct the benefits they receive from the desert, we conducted structured interviews with community members individually (Fig. 4). Our intention was to qualitatively explore what and how environmental stakeholders perceive, access, and use the ES provided by desert drylands.

In the structured interviews, the first question asked the respondents to identify, in their own terms, the benefits, both material and spiritual, that they derive from arid desert region. The investigator did not explain and define ES to the respondents, but simply translated the question in the structured interview into “benefits from the surrounding environment that enable the respondents to survive and live here and carry out their productive activities." Our goal was to enable interviewees to self-express how they benefit from the desert, rather than suggesting to them how they might benefit. The structured interviews were therefore open-ended, which may help to complement some of the shortcomings of economic valuation of ES. Then, to assess the importance of desert ES to environmental stakeholders, as well as their needs and preferences. Respondents were asked to select five benefits from the environment that they considered most important for their productive lives in the desert and to rank them on a scale of importance from 5 to 1 (where 5 is the highest and 1 is the lowest), as well as their reasons for making this choice. Finally, respondents were asked to rate these changes in availability from benefits, (5 significant increase, 4 slight increase, 3 no change, 2 slight decrease, 1 significant decrease), to understand the perception of changes in the availability of ES by environmental stakeholders under policies and management of land use change.

All conversations from the structured interviews were recorded for transcription and then analyzed for data. Here, we analyzed three questions from the interviews (i) what benefits do you derive from the environment and how do you access and use these benefits, (ii) which of these benefits derived from the environment is the most important to you and the reasons why it is important, and (iii) what are the changes in the perceived availability of these benefits derived from the environment after the land use change. We used the MEA categorization categories of ES, i.e., provisioning services, regulating and supporting services, and cultural services, the four main categories of as a basic framework. Deductive generalization of the interviews continued until no new categories and attributes of ES emerged. Once conceptual saturation was reached, we shifted our attention to behavioral patterns that could be interpretations of interviewees' preferences for how ES are accessed and used, and the impact of land use change policies and management on es availability. Through this case study in Northwest China, we aim to complement the methodology to the valuation of ES. Our aim is to try to use social science research methods to address how to value ES from the perspective of environmental stakeholders, and to provide information references for local land management and environmentally sustainable development policy making.

## 3 Results

### 3.1 Ecosystem services identification

A total of 28 ESs were identified by the two groups with different livelihood strategies (Table 2), of which provisioning services (8), supporting and regulating services (10) and cultural services (10). All respondents mentioned water and block wind and sand, all PPGs mentioned fodder and production area, and all APGs mentioned soil formation and conservation.

**Table 2:** Desert ecosystem services were identified by two groups.

	Themes	PPG	APG
Provisioning services	Water	100.00 %	100.00 %
	Food	98.36 %	91.04 %
	Firewood	96.72 %	85.07 %
	Fodder	100.00 %	76.12 %
	Construction materials	85.25 %	67.16 %
	Raw materials	70.49 %	76.12 %
	Medicinal herbs	95.08 %	95.52 %
	Minerals	85.25 %	85.07 %
Regulating/Supporting services	Block wind and sand	100.00 %	100.00 %
	Soil formation and conservation	90.16 %	100.00 %
	Climate regulation	96.72 %	94.03 %
	Reduce temperature by shade	91.80 %	86.57 %
	Shelter for people	93.44 %	79.10 %
	Production area	100.00 %	92.54 %
	Habitat for wildlife	98.36 %	98.51 %
	Drainage flood	98.36 %	76.12 %
	Improve soil	55.74 %	86.57 %
	Food safety	98.36 %	47.76 %
Cultural services	Aesthetic value of the landscape	55.74 %	77.61 %
	Sense of belonging	96.72 %	91.04 %
	Link to ancestors	81.97 %	94.03 %
	Recreation and entertainment	77.05 %	88.06 %
	Ecotourism	52.46 %	85.07 %
	Venue for the ceremony	93.44 %	95.52 %
	Cultural heritage value	93.44 %	68.66 %
	Meaningful Locations	91.80 %	47.76 %
	Growing memories	98.36 %	82.09 %
	Mental relaxation	96.72 %	61.19 %

*Percentage represents the proportion of people who identified the theme in this group. For PPG% = a/61, APG% = b/67, a and b represent the number of people in different groups who can recognize the theme.

More than 90 % of respondents also mentioned food and medicinal herbs in provisioning services, soil formation and conservation, climate regulation, production area and habitat for wildlife in supporting and regulating services, and the sense of belonging brought by a former home and venues where important ceremonies are held in cultural services. In contrast, the level of group perception was low for some ES, with less than one-half of APGs aware of food safety and meaningful locations.

A higher percentage of residents in the PPG group mentioned fodder, drainage flood, food safety, cultural heritage values, meaningful locations and making the spirit feel relaxed. And improved soil, aesthetic value of the landscape and ecotourism were recognized by more APGs.

### 3.2 Ecosystem service importance ranking

The two groups were asked to select and prioritize five of the identified “benefits from the desert” which important to them. The ES were prioritized in the following order of importance to the survival and well-being of the respondents (Table 3).

**Table 3:** Ranking the importance of desert ecosystem service.

PPG		APG	
Themes	Score	Themes	Score
Water	301	Water	331
Fodder	233	Soil formation and conservation	98
Production area	185	Block wind and sand	83
Sense of belonging	40	Climate regulation	83
Block wind and sand	31	Sense of belonging	63
Link to ancestors	22	Aesthetic value of the landscape	54
Medicinal herbs	20	Improve soil	49
Climate regulation	17	Link to ancestors	42
Reduce temperature by shade	14	Medicinal herbs	32
Cultural heritage value	12	Habitat for Wildlife	28
Mental relaxation	10	Ecotourism	23
Habitat for Wildlife	8	Reduce temperature by shade	22
Shelter for people	6	Recreation and entertainment	15
Venue for the ceremony	6	Cultural heritage value	15
Ecotourism	5	Fodder	14
Meaningful Locations	2	Meaningful Locations	13
Growing memories	2	Production area	12
Food safety	1	Venue for the ceremony	10
		Growing memories	10
		Food	4
		Shelter for people	4

Both groups selected water provisioning as the most important ES. From the results of the scoring of the importance of ES, PPG preferred provisioning services because their livelihoods depend heavily on the natural resources directly provided by the desert environment. More than 80 % of PPG respondents indicated that water, fodder, and production area are essential to their livelihoods, while APG preferred supporting and regulating services, including soil formation and conservation, block wind and sand, and climate regulation.

As for the choices of the types of ES of importance, PPG chose a total of three provisioning services, seven supporting and regulating services, and eight cultural services. Almost the same is true for the APG, where cultural services accounted for almost half of the options. Of the 10 cultural services identified, eight were selected as important by the PPG, while nine were selected by the APG.

Even though many ESs are considered important by local community residents, the choice of individuals and even groups to prioritize important ESs depends primarily on whether the ES is closely related to their livelihoods and, to a lesser extent, cultural needs.

### 3.3 Perceived changes in the availability of ecosystem services

Two groups with different livelihood strategies were given the option of choosing their perceptions of changes in the availability of desert ES over this twenty-year period after land use change. Statistical table of the PPG group's perceived evaluation of changes in the availability of ES (e.g., Table 4) Respondents in the PPG group perceived that the availability of the vast majority of provisioning services had decreased to varying degrees over the twenty-year period. More than 90 % of the PPG respondents perceived a decrease in water, raw materials for weaving and medicinal herbs. Of these, 78.69 % of PPG respondents believe that medicinal herbs have decreased significantly during this period. More than 50 % of PPG respondents felt that water and fodder also decreased significantly. In support and conditioning services, 81.97 % of PPG respondents perceived a decrease in habitat for wildlife, and nearly 50 % of respondents perceived a decrease in production area, and food safety.

**Table 4:** PPG's perceived evaluation of changes in the availability of ecosystem services.

Themes	Significant Increase	Slight Increase	No Change	Slight Decrease	Significant Decrease
Water	0.00 %	0.00 %	6.56 %	37.70 %	55.74 %
Food	4.92 %	9.84 %	27.87 %	21.31 %	36.07 %
Firewood	0.00 %	4.92 %	19.67 %	45.90 %	29.51 %
Fodder	3.28 %	3.28 %	13.11 %	29.51 %	50.82 %
Construction materials	0.00 %	13.11 %	54.10 %	18.03 %	14.75 %
Raw materials	0.00 %	0.00 %	6.56 %	52.46 %	40.98 %
Medicinal herbs	0.00 %	0.00 %	1.64 %	19.67 %	78.69 %
Minerals	0.00 %	0.00 %	29.51 %	44.26 %	26.23 %
Block wind and sand	34.43 %	22.95 %	8.20 %	14.75 %	19.67 %
Soil formation and conservation	11.48 %	18.03 %	39.34 %	18.03 %	13.11 %
Climate regulation	27.87 %	37.70 %	11.48 %	14.75 %	8.20 %
Reduce temperature by shade	4.92 %	44.26 %	18.03 %	21.31 %	11.48 %
Shelter for people	4.92 %	14.75 %	55.74 %	13.11 %	11.48 %
Production area	13.11 %	27.87 %	8.20 %	44.26 %	6.56 %
Habitat for Wildlife	0.00 %	0.00 %	18.03 %	50.82 %	31.15 %
Drainage flood	13.11 %	19.67 %	44.26 %	13.11 %	9.84 %
Improve soil	14.75 %	16.39 %	39.34 %	19.67 %	9.84 %
Food safety	0.00 %	3.28 %	45.90 %	37.70 %	13.11 %
Aesthetic value of the landscape	6.56 %	11.48 %	39.34 %	14.75 %	27.87 %
Sense of belonging	19.67 %	22.95 %	36.07 %	13.11 %	8.20 %
Link to ancestors	14.75 %	24.59 %	42.62 %	14.75 %	3.28 %
Recreation and entertainment	14.75 %	42.62 %	22.95 %	13.11 %	6.56 %
Ecotourism	11.48 %	27.87 %	32.79 %	13.11 %	14.75 %
Venue for the ceremony	8.20 %	19.67 %	27.87 %	29.51 %	14.75 %
Cultural heritage value	6.56 %	34.43 %	26.23 %	21.31 %	11.48 %
Meaningful Locations	14.75 %	21.31 %	39.34 %	14.75 %	9.84 %
Growing memories	13.11 %	18.03 %	37.70 %	19.67 %	11.48 %
Mental relaxation	8.20 %	18.03 %	29.51 %	27.87 %	16.39 %

Only three ES were perceived as having increased availability by more than 50 % of PPG respondents: block wind and sand, climate regulation and recreation and entertainment.

As can be seen from the statistical table of APG's evaluation of the perceived availability of desert ES (Table 5), reductions in provisioning services were agreed upon by both groups, with 62.69 % of APG respondents believing that there was a significant decrease in medicinal herbs that the desert could provide, and 55.22 % and 61.19 % believing that there was a decrease in water and minerals, respectively. The most striking characteristic of the APG group was that more than 50 % of the respondents perceived a decrease in the vast majority of cultural services, and the ES that have increased are all concentrated in supporting and regulating services.

**Table 5:** APG's perceived evaluation of changes in the availability of ecosystem services.

Themes	Significant Increase	Slight Increase	No Change	Slight Decrease	Significant Decrease
Water	2.99 %	19.40 %	22.39 %	25.37 %	29.85 %
Food	8.96 %	20.90 %	40.30 %	25.37 %	4.48 %
Firewood	5.97 %	13.43 %	50.75 %	20.90 %	8.96 %
Fodder	7.46 %	16.42 %	28.36 %	17.91 %	29.85 %
Construction materials	2.99 %	10.45 %	40.30 %	25.37 %	20.90 %
Raw materials	0.00 %	0.00 %	52.24 %	26.87 %	20.90 %
Medicinal herbs	0.00 %	0.00 %	17.91 %	19.40 %	62.69 %
Minerals	0.00 %	2.99 %	35.82 %	37.31 %	23.88 %
Block wind and sand	26.87 %	34.33 %	8.96 %	17.91 %	11.94 %
Soil formation and conservation	25.37 %	40.30 %	26.87 %	2.99 %	4.48 %
Climate regulation	10.45 %	13.43 %	31.34 %	40.30 %	4.48 %
Reduce temperature by shade	8.96 %	34.33 %	26.87 %	23.88 %	5.97 %
Shelter for people	13.43 %	20.90 %	32.84 %	26.87 %	5.97 %
Production area	16.42 %	40.30 %	31.34 %	7.46 %	4.48 %
Habitat for Wildlife	2.99 %	10.45 %	19.40 %	40.30 %	26.87 %
Drainage flood	0.00 %	35.82 %	40.30 %	16.42 %	7.46 %
Improve soil	26.87 %	40.30 %	20.90 %	8.96 %	2.99 %
Food safety	38.81 %	32.84 %	23.88 %	4.48 %	0.00 %
Aesthetic value of the landscape	8.96 %	25.37 %	26.87 %	16.42 %	22.39 %
Sense of belonging	5.97 %	16.42 %	13.43 %	26.87 %	37.31 %
Link to ancestors	2.99 %	7.46 %	22.39 %	34.33 %	32.84 %
Recreation and entertainment	16.42 %	25.37 %	20.90 %	22.39 %	14.93 %
Ecotourism	13.43 %	34.33 %	16.42 %	25.37 %	10.45 %
Venue for the ceremony	2.99 %	11.94 %	34.33 %	31.34 %	19.40 %
Cultural heritage value	13.43 %	14.93 %	20.90 %	34.33 %	16.42 %
Meaningful Locations	8.96 %	16.42 %	14.93 %	40.30 %	19.40 %
Growing memories	5.97 %	10.45 %	31.34 %	34.33 %	17.91 %
Mental relaxation	10.45 %	13.43 %	23.88 %	31.34 %	20.90 %

Perceived changes in the availability of desert ES also reflect a high level of group demand for a particular ES. Perceived reductions in it because the need is not being met, some reductions are objective facts while others are subjective perceptions. For example, before the change in land use, all villagers relied on a small amount of well water and springs to meet their production and living needs. Now the number of underground wells has increased a hundredfold to satisfy the irrigation of farmland. As far as water consumption is concerned, water is far increased, but both groups perceive a significant decrease in water. In addition to the disappearance and reduction of surface water, the deeper reason is the group's concern about the future availability of desert water sources.

50.82 % of the PPG respondents indicated that production area has decreased, while 56.72 % of the APG respondents indicated that production area has increased. This implies that there are trade-offs between pastoral production and agricultural development in the community regarding the use of desert space. These trade-offs can be attributed to the dominance of government policies, changes in natural conditions and choices made by community residents.

The results of this survey show that two groups with different livelihood strategies, PPG and APG, have different needs for ES in the desert, with PPG consistently showing more emphasis on ES that are closely related to their livelihoods, while APG, whose production activities are beginning to move away from the desert, have higher expectations of cultural services. They need more cultural services in the desert to access and strengthen their cultural and emotional connection to the desert.

## 4 Discussion

### 4.1 Perceptions of desert ecosystem services

Increased knowledge of ESs can enhance the importance that management places on the protection and management of desert ESs. In addition, increasing local people's knowledge of ES is also crucial, as some of the ES they are enjoying are not directly recognizable to community members, and it is only when they are aware of it that they can take it upon themselves to protect that ES from damage. Our study was able to understand local perceptions and preferences for ES, reveal different patterns of group ES acquisition and utilization, and understand these hidden influences.

The findings show that both groups with different livelihood strategies pay more attention to ES programs that are closely related to their production activities. The PPG emphasizes ES such as fodder, reduce temperature by shade, and production area, which are directly related to pastoral activities, while the APG is more concerned with services that contribute to agricultural production, such as soil formation and conservation. At the same time, PPG showed a stronger attitude in the results on the perception of changes in the availability of ES. The perception of change in availability emphasizes the subjective feelings of local people, which are both perceptions of the current situation and judgements of future trends. Due to the differences in labor intensity of females in different livelihoods, they are able to undertake the same production content as males in pastoral labor, whereas in agriculture the main labor force is male. Thus, women in APG tend to have neutral perceptions of ES change, while women in PPG show the strongest perceptions of change, expressing great concern about the curtailment of ES.

Second, the increased availability of supporting and regulating services is perceived uniformly by both groups. After the land use change, large numbers of Aspen trees were planted around residential settlements to block wind and sand. At the same time, windbreaks were planted in large numbers along the border between the desert and farmland to protect crops. Twenty years later the scale of the windbreak forests has been realized, and the function of ES that effectively block wind and sand, regulate the climate, and prevent soil erosion has increased. Diverting water to water trees near houses and farmland became the default responsibility of local residents, and cutting down and destroying trees was prohibited and restricted. Finally, both groups emphasized the importance of cultural services. This is inextricably linked to their traditional practices and culture. Cultural services are the result of a complex dynamic process of change between human societies and ecosystems over long periods of time. Cultural services are closely related to human values, modes of action and social organization. Because of the difference in livelihood strategies, women in APG value the leisure and recreation functions of cultural services and perceive an increase in them. On the other hand, women in PPG show a stronger cultural and local identity, they value the sense of place and homeland in cultural services and perceive a decrease in related cultural services.

### 4.2 Livelihood trade-offs and sustainability

Guaranteeing the sustainability of desert ES requires the attention and participation of local populations and cannot rely solely on government policy and project support. When ES fail to meet the livelihood needs of herders and farmers, the dynamic mechanism of ecological conservation is degraded.

**Fig. 5:**
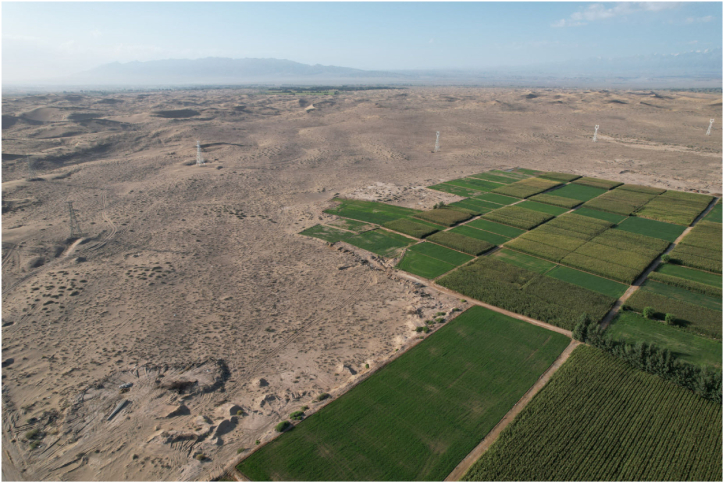
The desert being reclaimed into farmland. (Taken: July 2023; by T.Q).

In this study, there is an urgent trade-off between the ES associated with the livelihood activities of both groups. Water is recognized as an important ES by both groups, is the ES that is perceived to be significantly reduced by the largest number of people and is the focus of conflict between the two groups to maintain normal production. Pastoralists blamed the large extraction of groundwater for agricultural production, which has led to a drop in the water table and an inability of plant roots in the desert to absorb water, deepening desertification. Farmers, on the other hand, say that water is critical to the productivity of their land and to the income from their farming operations. The significant increase in water consumption is because the size of farmland is not being controlled and the total amount of groundwater should be measured before determining the size of farmland. Now, because there is more and more farmland and the water table has dropped so much, they are also forced to drill deeper wells to irrigate their farmland. If they are unable to continue farming because of water shortages, they will also lose their important livelihood. On the other hand, regarding the perception of changes in the availability of production area the two groups showed completely different perceptual results. The development of large amounts of desert grasslands for agriculture has greatly increased water consumption while squeezing pastoral production sites (Fig. 5). Most people do not consider desert areas to be ideal for agricultural production, a judgment that was made by ancestors who migrated here hundreds of years ago in their choice of livelihoods. For the future sustainability of the region, there is a need to find a balance between agricultural production and the protection of desert ecology, to resolve conflicts between habitat for wildlife, pastoral production and agricultural needs, to ensure a living environment for rare or sensitive species, and to restore and increase levels of biodiversity and ES.

Water resources are important for sustaining the living environment of all living organisms, but with population growth, climate change and changes in livelihoods and lifestyles, the pressure on water supplies is increasing, which increases the cost of production and ecological risks in arid areas. Numerous suggestions have been made and adopted to address the problem of water stress, for example, the government should vigorously implement water conservation measures and advocate drip irrigation and watering for every household; it should also carry out rigorous scientific planning for water resource management and measure the reasonable area of farmland in the region based on the total amount of groundwater; and prohibit the private reclamation of wasteland, the drilling of wells, and the blind expansion of agricultural production. However, to solve the problem of conflicting supply and demand of ecosystem services, it is not enough to increase the production of a certain type of ecosystem service to meet the current demand. At the centre of sustaining the productive lives of local populations is not depleting or damaging ecosystems without causing harm to the livelihoods and well-being of others or future generations. Overexploitation of ecosystems to meet present needs may temporarily increase people's material well-being and reduce their poverty, but such overexploitation will not be sustainable in the future. Therefore, in order to maintain the sustainable provisioning capacity of ecosystems, there is also a need to consider the trade-offs between current needs and future development. There is also a need to advocate and encourage sustainable agricultural intensification, which can help increase agricultural production without having to increase land area. On the other hand, desert ES can be maintained and restored by improving the quality of desert grasslands. Multi-systematic use of desert land is encouraged, such as the cultivation of medicinal herbs and the development of ecotourism as alternative livelihoods.

### 4.3 Cultural identity and place attachment

**Fig. 6:**
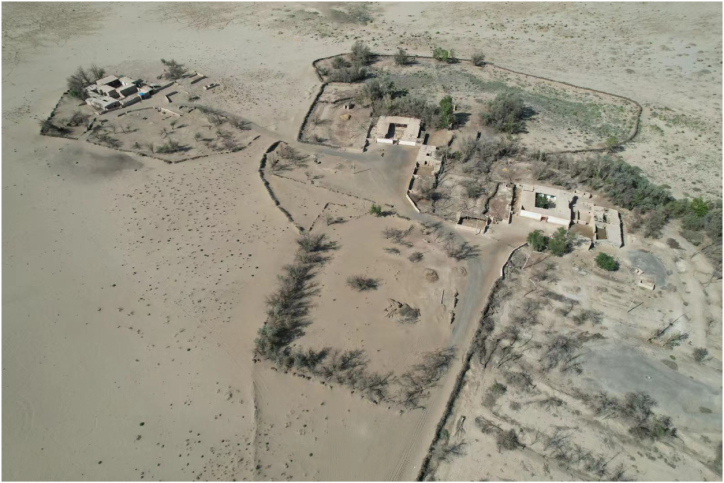
The “former home” of the Yugurs in the arid pastures of the desert.. (Taken: Aug. 2023; by T.Q.)

Cultural services build on the strong connection between people and ecosystems and are the most recent approach to explaining the relationship between humans and ecosystems and one of the most important tools for their protection. In the study, APG emphasized the importance and reduced availability of cultural services, which is closely related to the changes in livelihood strategies due to land use changes. With the changes in livelihood strategies, a series of changes in their living environment, social relations and traditional culture have made this group deeply aware of the significance of the previous way of life and place of production for their identity, and therefore show more direct and strong local attachment to their former homes than the pastoralists. More than half of the interviewees identified the houses in the desert simply as “former homes” (Fig. 6), expressing a deep identification with the place where they were born and grew up, and emphasizing the importance of this place in the formation of their personal identity and its symbolic significance. Therefore, it is only through uninterrupted contact with these specific places and interaction with nature that they can benefit from cultural services and satisfy their cultural and identity needs.

**Fig. 7:**
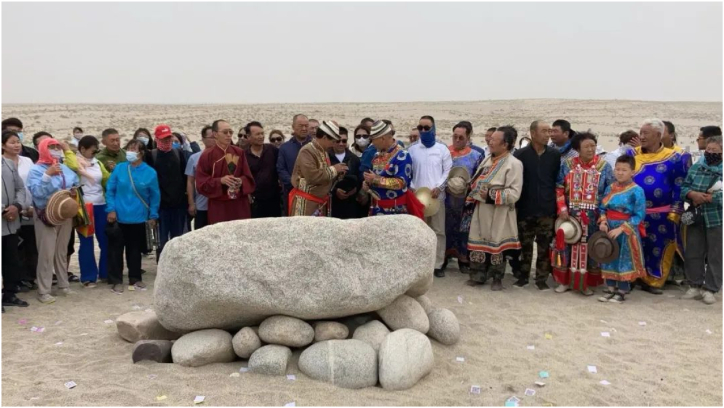
The Yugurs organize annual ceremonies in the desert to commemorate their ancestors who migrated here hundreds of years ago after a difficult journey. (Taken: July 2021; by L.WY.).

We further found that herding is the traditional livelihood of the Yugurs, and that the livelihood in a particular environment has also given birth to the nomadic culture of the Yugurs. Water, fodder, pasture, and shelter for livestock allow the Yugurs to continue their traditional pastoral livelihoods, and in this way, they safeguard the traditional culture of their people. Although local managers have been encouraging local people to reduce their resource dependence on natural ecosystems to ameliorate ecological problems. However, they have not considered the fact that even if the means of production and livelihood activities are removed from the natural environment of the desert, the local population still maintains a cultural and identity link with the desert (Fig. 7). If the desert ecosystem continues to deteriorate, the herders' livelihoods will be forced to change, and with it the nomadic culture and identity of the Yugurs may disappear.

Our analysis also supports the argument that traditional approaches to ES valuation can mask risks in spatial, historical, cultural, and economic trade-off processes in the study area, especially among different types of environmental stakeholders, and that psychological and cultural needs are ignored.

### 4.4 Ecosystem services and sustainable development

The overexploitation and overutilization of many natural resources to meet the survival needs of a growing global population has led to various forms of ecosystem degradation, such as reduced biodiversity, decreased productivity, and environmental pollution, which seriously threaten the sustainable development of human societies. However, ecosystem degradation does not necessarily determine the decline of ES completely. This is because ES may also be associated with increased demand or restrictions imposed by environmental regulations. Therefore, the use of ES as a framework for evaluating the increase or decrease of population well-being and sustainable development in a region better reflects the fact that the goal and focus of sustainable development is gradually shifting from single-ecosystem restoration to a multi-objective synergy of multiple ES enhancement and high-quality socio-economic development.

We therefore recommend that, as described in this study, sustainable development policies should be formulated with an understanding of which ES are important and prioritized for local communities and residents in the first place. However, economic valuation methods based on ES alone may not capture the nuances in how ES are perceived, accessed, and used by local people. In contrast, using a social science approach to ES valuation reveals the value of ES based on the different benefits they provide to local populations. This valuation approach is advantageous because it identifies ES that are commonly perceived and prioritized by local populations.

Incorporating the valuation of ES into decision-making processes can also help to raise awareness of the contribution of ES to human societies, as well as to generate positive responses and actions for ecosystem management, natural resource conservation, land and soil management, disaster management and water resource protection, and to improve regulatory mechanisms that strengthen the balance between conservation and sustainable development policies. This will prevent any poor decision-making that reduces the value of ES and help to improve local ES through continuous monitoring.

Our results can identify gaps between current policies and the needs of local people. Local communities can use local knowledge to report to policy makers on the needs and values of ES with which they are unfamiliar, and to suggest revisions to current land use, resource conservation policies. This bottom-up approach is simple, but the information is critical to developing management plans that promote ES conservation and sustainable regional development, and to safeguarding the effectiveness of plan and policy implementation.

## 5 Conclusions

We believe that maintaining the sustainable supply capacity of ES requires, first and foremost, consideration of the reconciliation and trade-offs between current needs and future development. At the center of maintaining sustainable regional development is not depleting or damaging ecosystems without causing harm to the livelihoods and well-being of others or future generations. Secondly, human well-being consists of multiple elements, including the right to equal access to ecosystem services, in addition to the material conditions that sustain life and production from ecosystems. Finally, cultural services are built on strong connections between people and ecosystems. People's perceptions of and preferences for cultural services shape particular environmental attitudes and behaviors. These cultural perceptions, in turn, have a significant impact on the health and safety of ecosystems and the harmony of social relations. We therefore advocate that land-use change and environmental protection policies and strategies need to take into account the livelihood, cultural and emotional interests of local people and minimize damage to their cultural and local ecological knowledge. Local sustainable development should be a long-term endogenous local development model, where the active participation and emotional support of environmental stakeholders is crucial to the success or failure of the development model. Incorporating the livelihood, cultural and emotional needs of environmental stakeholders into the consideration of the local sustainable development model can help to strike a balance between development goals and the needs of local residents, reduce conflicts between ecological environment protection and socio-economic development, and protect and enhance the well-being of local people.

This study uses the social science research method of participant observation and interviews to understand the value of local ES from the perspective of environmental stakeholders, which complements previous methods of ES valuation. At the same time, traditional values and traditional knowledge are integrated into the modern evaluation and decision-making framework, which not only further enhances the explanatory power of the relationship between people and ecosystems in ecological ethnology, but also expands the value of the practical application of ecological ethnology, which is precisely the academic significance of this study. However, there are some limitations to this study, i.e. only some of the opinions of elders involved in agriculture or pastoralism were explored in the selection of interviewees. It may be possible to increase the sample size of respondents and to consider and incorporate the views of more young people and other occupational groups in the planning of future development projects and the formulation of sustainable development strategies in the region.

## Ethics Declarations

For interviews, participatory, or observational research involving privacy, verbal or written informed consent to participate has been obtained from all participants prior the conduct of the study.

## Data availability statement

Data will be made available on request.

## Funding statement

This research was supported by Key Project of the National Social Science Foundation of China, entitled “Research on the Cultural Basis of the Interaction, Exchange and Integration of the Ethnic Groups in the Qilian Mountains" No. (21AMZ006).

## CRediT authorship contribution statement

**Qi Tan:** Writing – review & editing, Writing – original draft, Methodology, Investigation, Formal analysis, Data curation, Conceptualization. **Siru A:** Methodology, Investigation, Formal analysis, Conceptualization. **Wenying Lang:** Resources, Investigation.

## Declaration of competing interest

The authors declare the following financial interests/personal relationships which may be considered as potential competing interests:Qi Tan reports financial support was provided by National Social Science Foundation of China,. If there are other authors, they declare that they have no known competing financial interests or personal relationships that could have appeared to influence the work reported in this paper.

## Appendix A Survey Outline

1 what benefits do you derive from the environment and how do you access and use these benefits?
2 which of these benefits derived from environment is the most important to you and the reasons why it is important?
3 what are the changes in the perceived availability of these benefits derived from the environment after the land use change?
