# Supplementary material for: Assessing local people's perceptions of ecosystem services to support land management plans in arid desert regions, northwest China
Source: Heliyon. 2024 Jan 26;10(3):e25302. doi: 10.1016/j.heliyon.2024.e25302 (PMC10844273; doi:10.1016/j.heliyon.2024.e25302)
Supplement: Multimedia component 1 [file mmc1.docx]

**Questionnaire**

Dear Sir/Madam:

Greetings! We are students from the Center for Studies of Ethnic Minorities in Northwest China, Lanzhou University, where we are conducting a survey on local residents' perceptions of environmental change. Your participation in this survey is completely voluntary.

We sincerely invite you to give your own answers to the following questions, there is no right answer as long as it can truly reflect your thoughts to achieve the purpose of the survey. All data collected in the survey will only be used for statistical analysis to understand the impact of ecological change on the rights and interests of local people. If you choose to participate in this survey, your personal details and other information will not be shared with anyone outside the research team. Nor will any of your personal information be published when the results of the study are published.

Your responses are very important to our research, so thank you again for your support!

1. Gender：

☐ Male ☐ Female

2. Age：

☐Below 50 years old ☐51-60 years old ☐ Above 61 years old

3. Education level：

☐ Below junior ☐Junior school ☐High school ☐Above high school

4. what benefits do you derive from the environment and how do you access and use these benefits?

5.which of these benefits derived from environment is the most important to you and the reasons why it is important?

6.what are the changes in the perceived availability of these benefits derived from the environment after the land use change? (5=significant increase, 4=slight increase, 3=no change, 2=slight decrease, 1=significant decrease)

| Themes | 5 | 4 | 3 | 2 | 1 |
| --- | --- | --- | --- | --- | --- |
| 1. The water I can get from the environment |  |  |  |  |  |
| 2. The food I can get from the environment |  |  |  |  |  |
| 3. The firewood I can get from the environment |  |  |  |  |  |
| 4. The fodder I can get from the environment |  |  |  |  |  |
| 5. The construction materials I can get from the environment |  |  |  |  |  |
| 6. The raw materials I can get from the environment |  |  |  |  |  |
| 7. The medicinal herbs I can get from the environment |  |  |  |  |  |
| 8. The minerals I can get from the environment |  |  |  |  |  |
| 9. I think the function of blocking wind and sand |  |  |  |  |  |
| 10. I think the function of soil formation and conservation |  |  |  |  |  |
| 11. I think the function of climate regulation |  |  |  |  |  |
| 12. I think the function of reduce temperature by shade |  |  |  |  |  |
| 13. I think the function of shelter for people |  |  |  |  |  |
| 14. I think the function of production area |  |  |  |  |  |
| 15. I think the function of habitat for wildlife |  |  |  |  |  |
| 16. I think the function of drainage flood |  |  |  |  |  |
| 17. I think the function of improve soil |  |  |  |  |  |
| 18. I think the function of food safety |  |  |  |  |  |
| 19.Aesthetic value of the landscape |  |  |  |  |  |
| 20. The function of sense of belonging |  |  |  |  |  |
| 21. The function of link to ancestors |  |  |  |  |  |
| 22. The function of recreation and entertainment |  |  |  |  |  |
| 23. The function of ecotourism |  |  |  |  |  |
| 24. The function of venue for the ceremony |  |  |  |  |  |
| 25. The function of cultural heritage value |  |  |  |  |  |
| 26. The function of meaningful locations I get from here |  |  |  |  |  |
| 27. The function of growing memories I get from here |  |  |  |  |  |
| 28. The function of mental relaxation I get from here |  |  |  |  |  |
